# Serum Nesfatin-1 Levels in Girls with Idiopathic Central Precocious Puberty

**DOI:** 10.4274/jcrpe.4677

**Published:** 2018-02-26

**Authors:** Ayça Altıncık, Oya Sayın

**Affiliations:** 1 Denizli State Hospital, Clinic of Pediatric Endocrinology, Denizli, Turkey; 2 Dokuz Eylül University Faculty of Medicine, Department of Biochemistry, İzmir, Turkey

**Keywords:** Leuprolide acetate, nesfatin-1, nucleobindin-2, precocious puberty

## Abstract

**Objective:**

Nesfatin-1, an anorexigenic neuropeptide, is expressed mainly in the central nervous system and in some peripheral tissues. The role of nesfatin-1 in energy balance has been investigated. Despite the suggestion of a role for nesfatin-1 in reproductive function, data are limited on the role of nesfatin-1 in human puberty.

**Methods:**

The aim of this study was to investigate the following: i) the role of nesfatin-1 in puberty, and ii) relationship between nesfatin-1 and anthropometric measurements and gonadotropin levels in girls with idiopathic central precocious puberty (CPP). Twenty-four girls with CPP (7.68±1.02 years) and 20 female, prepubertal, healthy controls (7.48±0.88 years) were enrolled in the study. All patients with CPP were treated by the intramuscular administration of leuprolide acetate at a daily dose of 3.75 mg for 28 days. Nesfatin-1 was measured before and during treatment.

**Results:**

There was no difference in serum nesfatin-1 levels in girls with CPP and healthy controls [5.67 (2.5-20.6) mmol/L and 5.75 (2.51-9.64) mmol/L], respectively. There was a negative correlation between nesfatin-1 levels and body weight and body mass index-standard deviation score (p=0.01, r=-0.83; p=0.025, r=-0.81, respectively). No correlation was found between nesfatin-1 and gonadotropin, estradiol levels, uterine length or endometrial thickness.

**Conclusion:**

The results of this study suggest that there are no differences between girls with CPP and healthy, prepubertal girls regarding nesfatin-1 levels.

## What is already known on this topic?

Nesfatin-1, a recently discovered anorexigenic neuropeptide, seems to play an important role in hypothalamic pathways regulating food intake and energy homeostasis. There are a few reports suggesting the possible role of nesfatin-1 in metabolic regulation of reproductive function and the gonadotropic axis.

## 

### What this study adds?

This is the first study investigating the role of nesfatin-1 in human puberty. Although the sample size of our study is too limited to make precise comments, we found no evidence to support the role of nesfatin-1 in the regulation of human puberty.

## Introduction

Nesfatin-1, an 82-amino acid product of the post-translational processing of nucleobindin-2 (NUCB2), was initially described as an anorexigenic neuropeptide (1). Widespread expression of NUCB2 mRNA has been demonstrated in the central nervous system, especially in several hypothalamic nuclei and in forebrain-hindbrain areas that integrate both energy balance and reproduction (2). Several studies reported the co-localization of nesfatin-1 immunoreactive neurons with other neurotransmitters (including pro-opiomelanocortin, a-melanocyte-stimulating hormone and neuropeptide Y) that regulate food intake and with pituitary hormones (thyrotropine-releasing, growth hormone-releasing and corticotropin-releasing hormones). Immunohistochemical studies indicate that nesfatin-1/NUCB2 protein is expressed in peripheral organs including the stomach, pancreas, testes and the pituitary gland (3,4,5). The role of nesfatin-1 at the onset of puberty has been investigated in a few experimental studies. These studies have revealed data demonsrating the link between the neuroendocrine control of puberty and energy reservoirs and also the closer distribution of nesfatin-1 neurons to the key areas which control reproduction (6,7,8,9,10,11). These studies also showed that NUCB2 mRNA expression in hypothalamus, pituitary and testis changed during the pubertal transition (9,10). Furthermore, intracerebroventricular injection of nesfatin-1 in rats induced a significant increase in the serum levels of gonadotropins, mainly luteinizing hormone (LH) (6,9). In summary, evidence from rodent studies suggested that nesfatin-1; i) plays a role in the onset of puberty and in maturation and ii) affects the gonadotropic axis, mainly via increased secretion of LH (6,7,8,9,10,11). There is a close relationship between the adequacy of energy stores, adipose tissue and onset of puberty in children. The hypothalamo-pituitary-gonadal (HPG) axis has a capacity to respond to metabolic cues from energy stores. The most studied adipocytokine regarding the relationship between adipose tissue and puberty is leptin, which is an anorexigenic adipokine. Leptin plays an important role in the maturation of the HPG axis, and in onset and regulation of puberty, particularly in girls. However, there is no study investigating the role of nesfatin-1 in human puberty. In light of these findings, we hypothesised higher serum nesfatin-1 levels in girls with central precocious puberty (CPP). In this study, we aimed to investigate; i) serum nesfatin-1 levels in girls with CPP and ii) the relationship of nesfatin-1 with anthropometric parameters and gonadotropin levels.

## Methods

Girls who received a diagnosis of idiopathic CPP were enrolled in the study. The main criteria for a CPP diagnosis were; i) onset of breast development (Tanner breast stage 2) before the age of 8, ii) progression of pubertal development during follow-up for at least six months, iii) accelerated linear growth and advancement of bone age (BA), and iv) a basal LH level of ≥1 IU/L or a peak LH >5 IU/L measured during an intravenous gonadotropin-releasing hormone (GnRH) stimulation test. The control group consisted of healthy, prepubertal girls (Tanner stage 1) of similar age and body mass index (BMI) to the CPP patients. None of the girls in the control group had acute or chronic illnesses nor used any kind of medication. Physical examination and anthropometric measurements of the patients were performed by the same clinician (A.A.). Pubertal staging of each subject was recorded according to the Tanner classification (12). Height was measured by a stadiometer and weight was measured by a calibrated scale. The BMI was calculated as weight divided by height squared (kg/m^2^) and the BMI-standard deviation score (SDS) was calculated by using Centers for Disease Control and Prevention charts (13).All patients with CPP were treated with an intramuscular injection of leuprolide acetate (LA) at a daily dose of 3.75 mg for 28 days. A single X-ray of the left hand and wrist was performed to evaluate BA in the CPP group and the assessment of BA was performed by a single clinician, according to Greulich and Pyle (14). Pituitary magnetic resonance investigation (MRI) was performed on all patients. Pelvic ultrasonography for the evaluation of ovarian and uterine measurements was performed in all required patients.

Early morning blood samples were taken after 12 hours of overnight fasting and were immediately centrifuged. Serum samples were collected in Eppendorf tubes and stored at -80 ºC until the day of analysis for nesfatin-1 levels. Early-morning basal serum levels of LH, follicle-stimulating hormone (FSH) and estradiol were measured in all patients. 

The GnRH stimulation test with gonadorelin acetate (LHRH Ferring^®^, Ferring Pharmaceuticals Inc., Tarrytown, New York) was performed between 8 a.m. and 8.30 a.m. on subjects with basal LH <1 IU/L (15). GnRH (0.1 mg/m^2^) was administered intravenously and samples for measuring FSH and LH were drawn at 20, 40, 60 and 90 minutes after the injection. A peak LH level of >5 IU/L was considered to be indicative of puberty (16). For the assessment of hormonal suppression, a repeat GnRH test (retest) was performed during the course of treatment. Retests were done three weeks after the third dose of LA. Peak LH levels of <2 IU/L were considered to be an adequate suppression of puberty (17).

Serum nesfatin-1 measurement was performed using a human nesfatin-1, enzyme-linked immunosorbent assay commercial kit (Sunred Biological Technology, catalog 32 No. 201-12-4341, limit of determination 0.2-35 mmol/L) as recommended by the manufacturer’s protocol [Sensitivity: 0.113 mmol/L; Intra-Assay: coefficient of variability (CV) <10%; Inter-Assay: CV <12%]. The study design complied with the Declaration of Helsinki. Patient enrollment was started after the approval of the Ethics Committee of Pamukkale University (approval number: 60116787-020/25027). Informed written consent was obtained from the parents.

### Statistical Analysis

Data were analyzed using SPSS 17.0 computer software (SPSS, Chicago, Illinois, USA). Variables were given as the median (range). A Wilcoxon (two-related sample) test was used to compare medians of pretreatment and post treatment nesfatin levels of girls with CPP. The Mann-Whitney U test was used to compare the medians of the study and control groups. Univariate correlation analysis was performed using the Spearman test. A p value of <0.05 was considered to be statistically significant.

## Results

The study group consisted of 24 girls with CPP and 20 healthy prepubertal controls. The clinical and laboratory characteristics of patients are summarized in Tables 1 and 2. The median age interquartile range (IQR) at the time of treatment was 8.24 (6.6-10) years. Five patients (20.8%) were at Tanner stage 2, 12 (50%) at stage 3 and seven (29.2%) at stage 4 of pubertal development at the time of treatment. Eight patients (33.3%) underwent menarche before the age of 9.5 years. Median IQR BA at the time of treatment was 11 (8.8-12) years. The nesfatin-1 level of the CPP group was slightly higher than that of the healthy control group; however, the difference was not significant [5.67 (2.5-20.6) and 5.75 (2.51-9.64) mmol/L, respectively, p=0.32]. Also, there was no difference in nesfatin-1 levels prior to or during treatment (Table 1). There was a negative correlation between nesfatin-1 and body weight, BMI-SDS (p=0.01, r=-0.83; p=0.025, r=-0.81, respectively). Nesfatin-1 was not correlated with basal LH, basal FSH, basal estradiol, stimulated peak LH, retest peak LH, uterine length, endometrial thickness or pubertal stage in the CPP group.

The pituitary MRI of all patients was normal.

## Discussion

There are only a few studies on the role of nesfatin-1 in the HPG axis. Increased expression of hypothalamic NUCB2/nesfatin-1 during pubertal transition has been reported in female rats (9). Additionally, the central administration of nesfatin-1 induced elevation of circulating gonadotropins, especially LH in rodents (9). García-Galiano et al (8) reported that the expression of nesfatin-1 in mature Leydig cells was under the control of pituitary LH secretion. In contrast to these studies, a suppressive effect of nesfatin-1 on the hypothalamo-pituitary-ovarian axis of goldfish and rats has been demonstrated (10,18). Intracerebroventricular injection of nesfatin-1 was reported to significantly decrease the expression of the hypothalamic genes for *GnRH,* kisspeptin (*Kiss-1*), and pituitary genes for *FSH*b, *LH*b (18). However, these experimental studies were performed on non-mammalian vertebrates. To date, only a few reports exist on the role of nesfatin-1 in the human HPG axis. Çatlı et al (19) reported that serum nesfatin-1 levels were higher in girls with premature thelarche compared to those in healthy prepubertal controls. However, these researchers did not find a correlation between nesfatin-1 and gonadotropin levels. In a study by Abaci et al (20), there was no difference in nesfatin-1 levels between pubertal and prepubertal obese children (1.2±1.2 ng/mL and 1.3±2.0 ng/mL, respectively). In contrast to this study, Anwar et al (21)reported that there was a rise in the nesfatin-1 level as the pubertal stage advanced in both obese and healthy children. In this study, we did not find a difference in nesfatin-1 levels in girls with CPP and healthy controls. Also, there was no correlation between nesfatin-1 and gonadotropin levels, estradiol and pubertal stage. Intra-individual comparison of nesfatin-1 levels (pretreatment and during treatment) was not different. This finding suggested that circulating levels of nesfatin-1 may not have a role in the maturation of the HPG axis in the human. It is not possible to measure the central concentration of nesfatin-1 in humans. Thus it is not possible to investigate in humans its reported central role suggested by animal studies. There is a controversy in the literature about the relationship between nesfatin-1 and body weight and BMI-SDS. A positive correlation was reported between serum nesfatin-1 and BMI-SDS in obese children in previous studies (21). Serum nesfatin-1 was found to be significantly higher in obese children than in control groups. Additionally, these same authors reported a positive correlation between serum nesfatin-1 with serum insulin, BMI-SDS, body fat % and fat mass. In addition Ustabaş Kahraman et al (22) have reported lower nesfatin-1 levels in underweight children compared to healthy controls. In contrast to these findings, Abaci et al (20) found a negative correlation between nesfatin-1 and BMI-SDS in obese children, and nesfatin-1 levels were lower in obese children than in healthy controls. It was speculated that lower levels of nesfatin-1 might be the reason for uncontrolled appetite in obese children. In the present study, we found a negative correlation between nesfatin-1 and BMI-SDS. However, obese children were excluded from our study group and median BMI-SDS was within the normal range in this group.

### Study Limitations

Our study has limitations. Firstly, we have a relatively small number of participants, so this study could be considered as a preliminary report. Secondly, indices of adiposity such as body composition, fat distribution or waist circumference measurements were not investigated in our study.

## Conclusion

In conclusion, the results of our study suggest that there are no differences in nesfatin-1 concentrations between girls with CPP and prepubertal girls. Further, larger scale studies, including those on the expression of nesfatin-1/NUCB2 in the pituitary and in gonads in humans with CPP, are needed to clarify this matter.

## Figures and Tables

**Table 1:**
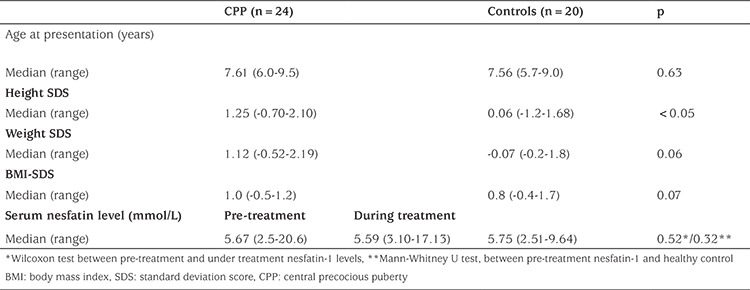
The clinical characteristics of patients with central precocious puberty and the control group

**Table 2:**
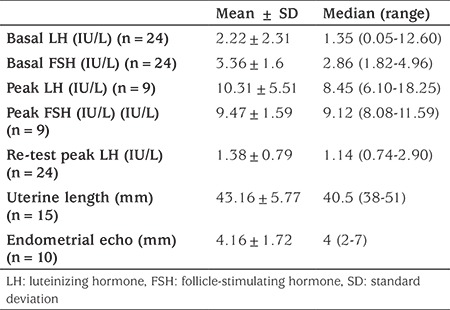
Biochemical and radiological characteristics of patients with central precocious puberty
